# Preparation and Characterization of Food-Grade Pickering Emulsions Stabilized with Chitosan-Phytic Acid-Cyclodextrin Nanoparticles

**DOI:** 10.3390/foods11030450

**Published:** 2022-02-03

**Authors:** Jiaxin Lu, Xiaojing Li, Chao Qiu, David Julian McClements, Aiquan Jiao, Jinpeng Wang, Zhengyu Jin

**Affiliations:** 1 State Key Laboratory of Food Science and Technology, School of Food Science and Technology, Synergetic Innovation Center of Food Safety and Nutrition, Jiangnan University, Wuxi 214122, China; 6190112071@stu.jiangnan.edu.cn (J.L.); q930017357@163.com (C.Q.); aqjiao@jiangnan.edu.cn (A.J.); 2 College of Light Industry and Food Engineering, Nanjing Forestry University, Nanjing 210037, China; lxj0810jy@163.com; 3 Department of Food Science, University of Massachusetts, Amherst, MA 01060, USA; mcclements@foodsci.umass.edu; 4 Beijing Advanced Innovation Center for Food Nutrition and Human Health, China-Canada Joint Lab of Food Nutrition and Health (Beijing), School of Food and Health, Beijing Technology and Business University (BTBU), 11 Fucheng Road, Beijing 100048, China; wangjinpeng1984@126.com

**Keywords:** chitosan/cyclodextrin, nanoparticles, Pickering emulsions, rheological properties, stability, phytic acid

## Abstract

This study aimed to fabricate food-grade Pickering emulsions stabilized by chitosan-phytic acid-β-cyclodextrin (CS-PA-CD) nanoparticles. The CS-PA-CD nanoparticles were characterized with FITR, XRD, and TGA to prove its successfully crosslinking, then characterized by DLS system and scanning electron microscopy showing the smallest average particle size was 434.2 ± 2.5 nm and it increased with the ratio of PA-CD to CS increasing. Pickering emulsions stabilized by CS-PA-CD nanoparticles was prepared and it showed the best stability at around pH 6. The particle concentration higher than 1.0% (*w*/*v*) and the oil fraction above 0.5% (*v*/*v*) could reach the emulsion stability. In addition, the Pickering emulsions were stable at various temperature (30–70 °C) and influenced by the certain change of ionic strength (0–500 mM). These CS-PA-CD Pickering emulsions showed great application in the formation of functional foods and pharmaceutical industries.

## 1. Introduction

Chitosan (CS) is a linear polysaccharide composed with repeated D-glucosamine and N-acetyl-D-glucosamine units, derived from the deacetylation of chitin [1]. It is a biopolymer with multiple properties—such as gelling, antibacterial, mucoadhesive, and antifungal—and these unique properties have made it been used in several applications like hydrogels, nanoparticles, microcapsules, and drug and bioactive delivery systems [2,3,4]. Among all these applications, the Pickering emulsions stabilized by chitosan-based nanoparticles has brought much attention because of the considerable demands of food-grade emulsifiers and its high stability against aggregation of Pickering emulsions [5,6,7]. Pickering emulsions are emulsions stabilized by solid particles instead of traditional emulsifier like surfactants or amphiphilic biopolymers. The first common method used to prepare chitosan nanoparticles is self-aggregation [8,9,10]. However, the former studies have found that chitosan is not a satisfactory emulsifier for preparing the O/W emulsions due to its hydrophilic properties. Therefore, it needs appropriate modification to improve emulsification.

The most used modification is hydrophobic modification, which usually happens between the amino groups of chitosan and the alkyl groups in the compounds of carboxyl groups, leading to the addition of a hydrophobic segment to chitosan to improve its emulsifying properties [1]. Atarian and Hosseini et al. fabricated chitosan-stearic acid nanogel mediated through EDC, then prepared the Pickering sunflower emulsions [11,12]. Yan, McClements et al. fabricated OSA starch/chitosan polysaccharide complex to stabilized high internal phase emulsion [13]. There are other methods to formulate chitosan-based particles. There are findings that the usage of mono-dispersed gliadin/chitosan hybrid particles with 588.8 nm size and wettability of 82.74° can be prepared HIPEs up to 83% internal phase with low particle concentrations [14]. It is noteworthy that hydrocaffeic acid modified chitosan crosslinking with TPP through the amidation reaction and ionic gelation can also be applied to stabilize the Pickering emulsions [15].

β-cyclodextrin (β-CD) is a cyclic oligosaccharide composed of seven D-glucopyranose units linked through 1,4-glucosidic bonds, coming from controlled enzymatic hydrolysis of starch [16,17]. Its hydrophobic cavity interior and hydrophilic cavity exterior make it soluble in water and capable for the encapsulation of hydrophobic ingredients [18,19]. Meanwhile, cyclodextrins have been widely used to prepare Pickering emulsions under rotor-stator homogenization, high-pressure homogenization, and sonication [20,21,22]. It was proved that the cyclodextrin contact angle with the oil/water interface defined the type of emulsions [23], and the stability of Pickering emulsions prepared by α- or β-CD were significantly higher than γ-CD [24]. However, due to the instability of Pickering emulsions stabilized by cyclodextrins, modification on native CDs became popular trends to improve their solubility and other functional properties [25], such as reacting with octenyl succinic anhydride (OSA) and synthesizing CD-based metal-organic frameworks [26,27].

Therefore, it is worthwhile to combine β-cyclodextrin and chitosan to fabricate nanoparticles, which can improve both disadvantages [28,29]. Previous studies have confirmed that tripolyphosphate-b-cyclodextrin (TPP-b-CD) can be used to reacting with chitosan to form nanoparticles, with smaller particle size compared to the traditional TPP-chitosan nanoparticles [30]. Sang also reported that CS-NPs crosslinked by phytic acid (PA) has a better encapsulation efficiency of myricetin and slower drug release rate compared with CS-NPs crosslinked by TPP [31]. There are some issues on the controversial nutrition aspect, like the intake of phytic acid may bringing undesirable effects on the bioavailability of minerals. However, it also does help in preventing the oxidative deterioration of food [32]. In this study, a novel chitosan/β-cyclodextrin nanoparticles was developed to prepare Pickering emulsions. Phytic acid was first used to modify β-CD by the dry-heating method [33], then reacting with chitosan through ionotropic gelation to obtain CS-PA-CD nanoparticles. The structural features of particles were explored by multiple analytical methods. Thereafter, Pickering emulsions stabilized by CS-PA-CD nanoparticles was characterized by droplet size, images, rheological measurements, and stability against environment factors to investigate its properties.

## 2. Materials and Methods

### 2.1. Materials

The phytic acid (≥70%), NaH_2_PO_2_, β-cyclodextrin (≥99.5%), acetic acid (≥99.5%), absolute ethanol (≥99.7%), NaOH (≥96.0%), and HCl were purchased from Sinopharm Chemical Reagent Co., Ltd. (Shanghai, China). Chitosan (<100 mPa•s) was obtained from Ryon Biological Technology Co., Ltd. (Shanghai, China). Nile Red was purchased from Aladdin Regent Co., Ltd. (Shanghai, China). Soybean oil was ordered from a local supermarket (Wuxi, China). All materials were used without further purification.

### 2.2. Preparation of CS-PA-CD Nanoparticles

#### 2.2.1. Preparation of Phytic Acid Modified β-Cyclodextrin

Phytic acid modified β-cyclodextrin was prepared according to the method previously reported [33,34]. Briefly, the beta-cyclodextrin (1 wt%) was dissolved in distilled water, followed by adding NaH_2_PO_2_ and phytic acid in ratios of 1:2. The solution was then dried in oven at 100 °C, after which the resulting dried mixture went through a treatment in a 130 °C oven for 20 min. The products were recovered after the addition of water to solubilize the reaction materials and washed with ethanol several times until not being sticky, then dried at 60 °C overnight. Thereafter, the PA-CD was stored in a dryer waiting for further analysis.

#### 2.2.2. Preparation of CS-PA-CD Nanoparticles

The preparation of CS-PA-CD nanoparticles was made based on the method of Wang, Qiu et al. [35]. CS was dispersed in acetic acid (2%, *v*/*v*) at a concentration of 1 wt% and stirring overnight to ensure dissolution at room temperature. PA-CD (1 wt%) obtained in previous step was dissolved in distilled water and then added into CS solution under constant stirring at different PA-CD:CS (*w*/*w*) ratios of 2:1, 1.5:1, 1:1, 1:1.5, 1:2. The system was reacted at 50 °C for 4 h. The CS-PA-CD nanoparticles dispersion was obtained. At the end of reaction, the solution was filtered and the supernatant was collected. Thereafter, ethanol in 10 times volume was added into the residues and the sediment was collected and washed by ethanol. Detailed information were found in Table 1. The particles were freeze-dried and kept in dryers for further analysis.

### 2.3. Characterization of CS-PA-CD Nanoparticles

#### 2.3.1. Physical Properties of CS-PA-CD Nanoparticles

As reported by Wang, Qiu et al. [35], in order to identify the changes in chemical structure of CS-PA-CD nanoparticles, pure beta-cyclodextrin, pure chitosan, phytic acid, and the different CS-PA-CD nanoparticles were analyzed in a Nicolet Nexus 470 Fourier transform infrared (FTIR) spectrometer (Thermo Electron Corporation, Waltham, MA, USA) through 400 to 4000 cm^−1^ scanning ranges. X-ray diffraction (XRD) analysis were performed using an X-ray diffractometer (D2 PHASER, Bruker, Karlsruhe, Germany) with an 4° to 40° scanning range of the diffraction angle of 2θ. The thermal stability of CS-PA-CD nanoparticles was assessed by the thermogravimetric analysis system (TGA2, Mettler–Toledo, Schwerzenbach, Switzerland).

#### 2.3.2. Particle Size of CS-PA-CD Nanoparticles

The particle size and polydispersity index (PDI) of CS-PA-CD nanoparticles were measured using Zetasizer Nano ZS90 (Malvern Instruments Ltd., Malvern, UK) after dispersing (0.1%, m/v) in distilled water. The morphology characterization of CS-PA-CD nanoparticles was performed by scanning electron microscope (SU8100, SEM, Hitachi, Tokyo, Japan) after the samples being coated with gold.

### 2.4. Preparation of Pickering Emulsions

The Pickering emulsions were prepared using different CS-PA-CD nanoparticles concentrations (0.1%, 0.5%, 1%, 1.5%, and 2%, *w*/*v*) and various oil phase fractions (45%, 50%, 55%, 60%, and 65%, *v*/*v*). The total volume of Pickering emulsions was fixed at 20 mL. The freeze-dried CS-PA-CD nanoparticles were redispersed into ultrapure water, then the soybean oil was added into the suspensions and emulsified together using a highspeed homogenizer (UltraTurrax Digital D-500, Wiggens, Germany) at 12,000 rpm for 3 min [36].

### 2.5. Droplet Size and Zeta Potential Measurements of Pickering Emulsions

The droplet size and zeta potential of Pickering emulsions stabilized by CS-PA-CD nanoparticles was determined by Malvern Zetasizer Nano (Malvern Panalytical, Ltd., Malvern, UK) through dynamic light scattering. All the measurements were taken at room temperature and at an angle of 90°.

### 2.6. Microscopy Analysis of Pickering Emulsions

The microstructure of Pickering emulsions was visualized using confocal laser scanning microscope (CLSM) (LSM880, Zeiss, Oberkochen, Germany). The oil phase was stained by Nile Red (0.1%) dye before emulsified with CS-PA-CD nanoparticles suspensions. The stained sample was placed on concave confocal microscope slides. The dye was excited by an argon laser at 488 nm and displayed in green.

### 2.7. Rheological Measurements of Pickering Emulsions

The rheology properties of Pickering emulsions were examined using a Discovery HR-3 Rheometer (TA Company, New Castle, DE, USA) at 25 °C with a plate geometry (40 mm diameter, gap 1 mm). The samples firstly underwent shear rates from 0.1 to 100 s^−1^ for the steady shear experiments, and the apparent viscosity (η) was recorded. Thereafter, the dynamic frequency sweeps were performed between 0.1–100 rad/s within the linear viscoelastic region under 0.1% strain, the storage modulus (G′) and loss modulus (G″) were determined.

### 2.8. Assessment of Emulsions Stability to Different Influencing Factors

In order to estimate the emulsion’s stability to different ionic strengths, an array amount of NaCl powder was added into freshly made emulsions of 10 mL (1% CS-PA-CD nanoparticles and 50% oil phase) in a concentration of 0–0.5 M. The thermal properties of Pickering emulsions were evaluated by heating emulsion samples to an array of temperature (30, 40, 50, 60, and 70 °C). A series of emulsion samples were prepared by 1% CS-PA-CD nanoparticles with 50% oil phase fraction and the pH was adjusted to 3, 4, 5, 6, 7, 8, and 9 using either HCl or NaOH, in order to evaluate the effects of pH values on Pickering emulsions stability.

### 2.9. Statistical Analysis

All measurements were conducted on sperate samples in triplicate and presented as mean values ± standard deviations. Variance (ANOVA) procedure of the SPSS 17.0 statistical software (SPSS Inc., Chicago, IL, USA) was used to analyze the experimental data. Differences were considered at a significance level of 95% (*p* < 0.05), and the activity order of different columns is a > b > c > d > e.

## 3. Results and Discussion

### 3.1. Characterization of CS-PA-CD Nanoparticles

#### 3.1.1. Physical Properties of CS-PA-CD Nanoparticles

In this study, the hydroxyl groups of beta-cyclodextrin crosslinked with the phosphate group of phytic acid first, then reaction between the remaining phosphate group of previously synthesized PA-CD and the amino groups of CS at different PA-CD to CS ratios of 2:1, 1.5:1, 1:1, 1:1.5, and 1:2.

**Figure 1:**
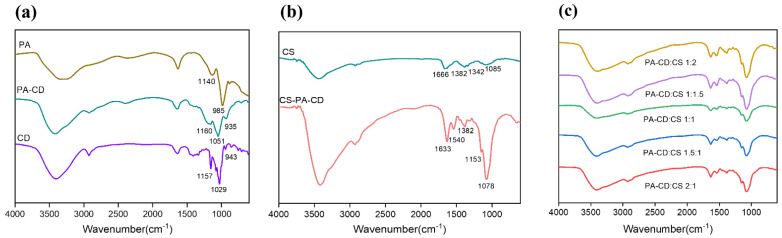
FTIR spectrum of (**a**) β-CD, PA, PA-CD, (**b**) CS and CS-PA-CD with (**c**) different ratios of PA-CD to CS.

The structure obtained by FTIR spectroscopy for β-CD, PA, PA-CD, CS, and CS-PA-CD were presented in Figure 1. In Figure 1a, the β-CD spectrum showed several characteristic peaks, a peak at 3401 cm^−1^ related to the stretching vibration of -OH groups and the peak at 2925 cm^−1^ associated with the stretching vibrations of -CH groups [37]. The rest peaks at 1411 cm^−1^ (-OH bending), 1157 cm^−1^ (C-O-C bending), and 1029 cm^−1^ (C-O-C stretching), were also characteristic peaks of β-CD. In the PA spectrum, the peak at 1140 cm^−1^ was attributed to the stretching vibrations of P=O groups, while at 985 cm^−1^ related to the P-O-C vibrations [38]. As shown in Figure 1a, the spectrum of PA-CD showed typical characteristic absorption peaks of CD at 1160 and 1051 cm^−1^, which can be attributed to the stretching of the C-O-C and the C-O groups. In addition, there were some characteristic peaks of PA disappeared revealed that β-CD was successfully chemically cross-linked to phytic acid [30].

In Figure 1b, the pure chitosan presented a peak at 1666 cm^−1^, which indicated the presence of secondary amino group (NH bending), a peak at 1085 cm^−1^ associated with C-O-C stretching and other characteristic peaks at 3440 cm^−1^ and 2874 cm^−1^, attributed to -OH and -CH stretching respectively [39]. In the spectrum of CS-PA-CD, the region of peak at 3417 cm^−1^ became deeper, proving the hydrogen bonding action strengthened and the peak of -NH_2_ bending vibration shifted from 1666 to 1633 cm^−1^ [39]. It was also noteworthy that a new peak appearing at 1540 cm^−1^ related to the interaction between PA and the NH_3_^+^ groups of CS. To sum up, the aforementioned factors led to the formation of CS-PA-CD nanoparticles. Figure 1c depicted the spectrums of CS-PA-CD nanoparticles at different CS to PA-CD ratios. It could be observed that the peak region of hydrogen bond became smaller as the decreasing portion of CS under 1:1 ratio, proving the hydrogen bond strength weaker due to the less amount of CS reacting with PA.

**Figure 2:**
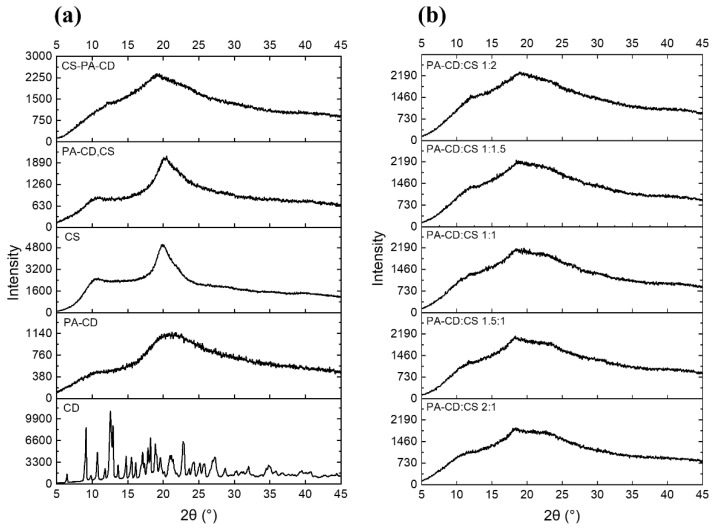
XRD patterns of (**a**) β-CD, PA-CD, CS, physical mixture of PA-CD and CS and (**b**) CS-PA-CD nanoparticles with different ratios.

The XRD patterns of different samples were presented in Figure 2. The diffractograms were obtained to assess the formation of PA-CD and CS-PA-CD nanoparticles. The diffraction peaks at 2θ of 10° and 21° were two characteristic peaks of PA-CD. Obviously, fewer peaks were obtained in the diffractogram of PA-CD compared to β-CD, indicating the reaction with phytic acid leading to the crystalline of beta-cyclodextrin decreased. The characteristic peaks of CS were at 11° and 20°, and there were no big differences between the patterns of CS and the physical mixture of PA-CD and CS, while in CS-PA-CD nanoparticles the peaks shifted to 12° and 19° with larger peak areas compared with CS patterns, revealing that the amorphous phase after complexation. The result was similar to what Hu et al. had found [30], where they found that there were diffraction peaks disappearing and the broader peak in the TPP-β-CD/CS nanoparticles confirmed that CS and TPP-β-CD both in an amorphous phase because of the crosslinking reaction. In Figure 2b, the characteristic peaks value became lower with the PA-CD:CS ratio higher, suggesting the addition of CS will affect the crystallinity degree.

**Figure 3:**
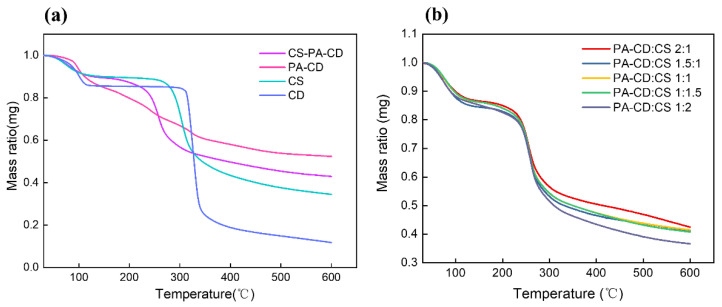
TGA curves of (**a**) different samples (PA-CD:CS, 1:1) and (**b**) CS-PA-CD nanoparticles with different ratios of PA-CD to CS.

The thermal stability of different samples was shown in Figure 3. The TGA curves of β-CD, CS, and CS-PA-CD nanoparticles generally showed a patterns of three-step weight losses (Figure 3a). The degradation taken placed in the region of 40 to 120 °C mainly corresponded to the dehydration of absorbed water, based on the result of Osman, Z. et al., who suggested that the weight loss in the range of 136 to 250 °C was attributed to the loss of acetic acid in the polymer, while there was no weight loss in our experiments [40]. The results showed that after modification, CS-PA-CD had about 10% moisture while pure beta-cyclodextrin had 14% moisture, revealing that CS-PA-CD nanoparticles had lower moisture and weaker water absorption capacity [41,42]. The degradation of pure beta-cyclodextrin started at 300.3 °C and CS started at 252.6 °C, while CS-PA-CD started at 181.8 °C, possibly associated with more hydroxyl groups considering that the main decomposition mechanism was through reaction between the hydroxyl groups. However, the residual mass of CS-PA-CD was higher than β-CD and CS while the residual mass of PA-CD was the highest, confirming the existence of PA and CS could elevate the thermal stability effectively [40,42]. According to the TGA result of Wang, Gao et al. [42], the residual mass of PA was 33%, while the residual mass of PA-GO (69.1%) was greater than that of GO (51.3%). They also ascribed this phenomenon to the modification of PA. The results indicated that nanoparticles have stronger structures than pure β-CD and CS, and have higher thermal stability due to the formation of phytic acid between cyclodextrin and chitosan. In Figure 3b, the first and second stages were similar in different ratios of PA-CD to CS, yet the residual mass at 600 °C decreased with the increased ratio of CS. This phenomenon indicated that the nanoparticles with lower partition of CS exhibited higher thermal stability and have relative stronger structures, due to the decomposition of chitosan starting at 250 °C as Osman, Z. et al. suggested [40].

#### 3.1.2. Average Size and Morphology of CS-PA-CD Nanoparticles

The size of CS-PA-CD nanoparticles measured by DLS with different ratios were shown in Table 2. It is clear that the particle size increase as the ratio of PA-CD to CS decreasing, as the mean diameter of nanoparticles increased from 434.2 nm for 2:1 ratio to 811.1 nm for 1:2 ratio. The phenomenon was similar to the result of Shah’s [10]. The findings mainly were associated with phosphate groups in phytic acid interacting with the amino groups of excess CS polymer through forming intermolecular and intramolecular crosslinking. All the PDI values were smaller than 0.250, indicating a narrow nanoparticles particle size distribution. Zeta potentials were also presented in Table 2. In all samples, the nanoparticles had positively charged surface. A gradual increase in the value of zeta potentials appeared with the increase in the PA-CD:CS mass ratio, which were related to residual amine groups in CS [28]. However, there was a slight increase from 11.18 mV of 1.5:1 ratio to 15.25 mV of 2:1 ratio, mainly due to the increasing amount of CS accompanying with the amine groups.

**Figure 4:**
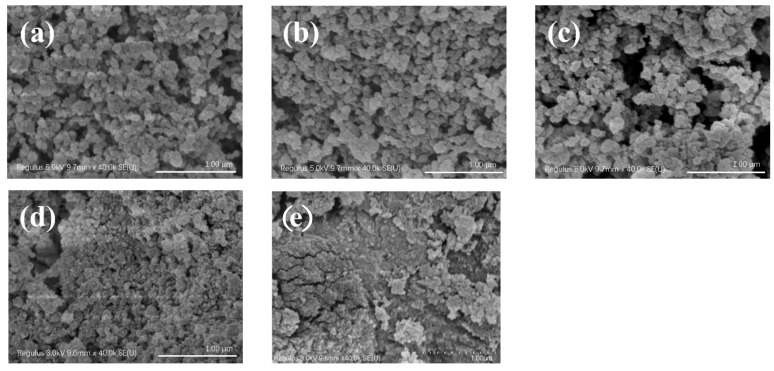
SEM images of CS-PA-CD nanoparticles with different ratios of PA-CD to CS: 2:1(**a**), 1.5:1(**b**), 1:1(**c**), 1:1.5(**d**), 1:2(**e**).

The morphological characteristics of CS-PA-CD nanoparticles were estimated using SEM. The SEM images (Figure 4) suggesting that CS-PA-CD nanoparticles were mainly spherical in shape and the mean particle size was under 500 nm, which was the same as the results of DLS measurements. However, when the ratio of PA-CD to CS was above 1:1.5, the particles tended to link each other and became gels. This phenomenon mainly was attributed to the high ratio of chitosan leading to more connection with the phosphate groups of phytic acid, thus causing the higher particle size.

#### 3.1.3. Zeta Potential on Different pH Values

**Figure 5:**
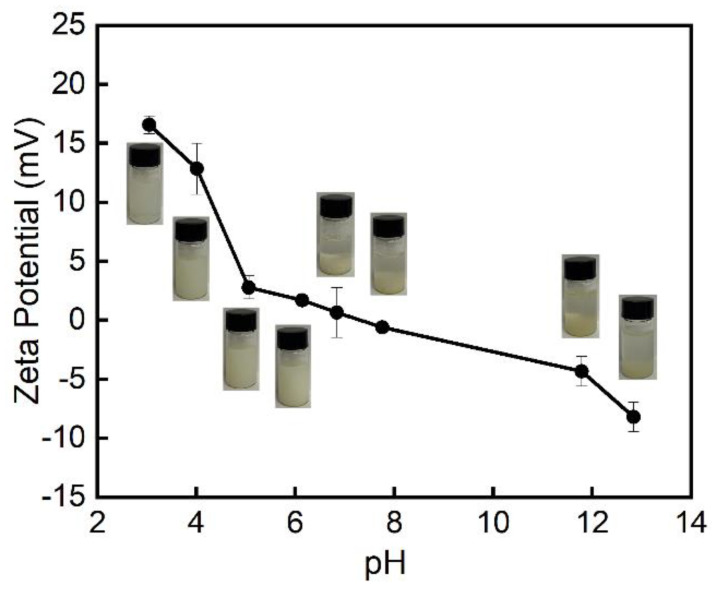
The zeta potential of CS-PA-CD nanoparticles on different pH values.

The pH of the CS-PA-CD dispersions was varied from 3 to 12. As can be seen in Figure 5, the zeta potential of nanoparticles showed a decrease decline with the increasing pH values, attributed to the amount of the amino groups on the chitosan. It shows a pKa at around 7. Furthermore, the CS-PA-CD dispersions below pH 6 were semitransparent and milky, and it became insoluble when the pH value above 6, showing the formation of CS-PA-CD nanoparticles sensitive to pH value due to the chitosan.

### 3.2. Characterization of Pickering Emulsions Stabilized with CS-PA-CD Nanoparticles

#### 3.2.1. Appearance and Droplet Size of Pickering Emulsions

**Figure 6:**
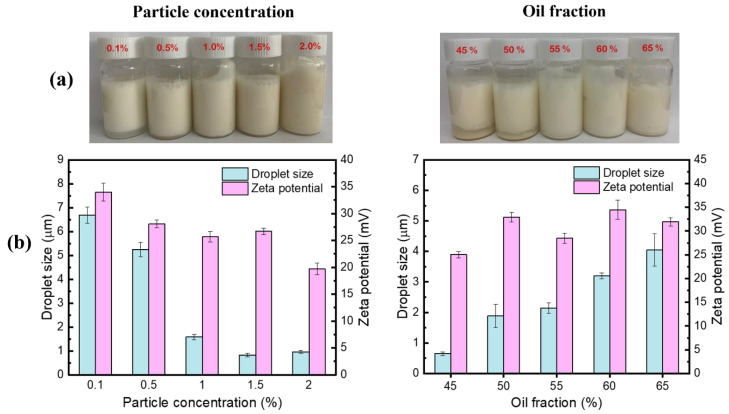
(**a**) Appearance, (**b**) droplet size and zeta potential of droplets of Pickering emulsions under different particle concentrations (fixed oil fractions: 50%) and oil fractions (fixed particle concentration: 1%).

**Figure 7:**
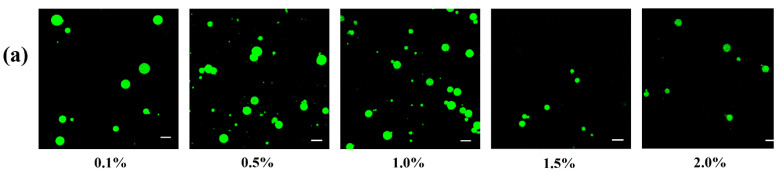

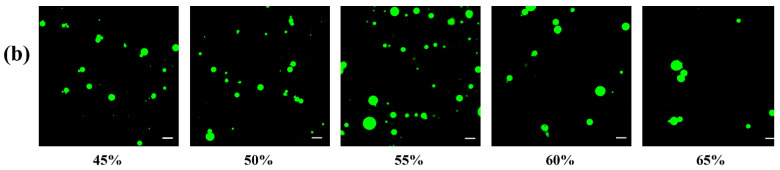
Morphology of Pickering emulsions stabilized with different particle concentrations (**a**) and oil fractions (**b**). The scale bar is 10μm.

Figure 6 showed the droplet size and visual appearance of Pickering emulsions stabilized by different nanoparticle concentrations of CS-PA-CD at the fixed oil fraction (0.5%). It is clear that emulsions exhibited increased stabilities and lower creaming index values with rising particle concentrations. The color of the emulsions became slightly deeper when the concentration was 2.0%, mainly due to the increasing number of nanoparticles. The droplet size of emulsions was decreasing with the higher particle concentrations, which was similar to the observation of Li, Xie et al. and Kiokias, Gordon et al. [36,43]. When oil fraction was fixed, higher particle concentration leading to higher particle thickness on the droplet interface, therefore could cover a larger interfacial area and the emulsion tend to have smaller droplets. When the particle concentration was beyond 1.0%, the creaming index value remained unchanged. Thus, the 1.0% particle concentration was selected for further analysis. The morphological structure of Pickering emulsions was observed using confocal laser scanning microscopy (CLSM) to gain more information (Figure 7a). The oil droplets were dyed by Nile red. Most of emulsion droplets were spherical shape evenly distributed, further confirmed that the emulsion droplets size decreased as the particle concentration increased. A decreasing trend of zeta potentials was presented in Figure 6b, same with the trend of droplet size. However, all the absolute zeta potential values (except 0.1% particle concentration) were below 30 mV, which is considered the required zeta potential value for the physical stability of Pickering emulsions [44], confirming that no correlation between the emulsion stability and the zeta potential values.

The droplet size of Pickering emulsions with different oil fractions at fixed particle concentration (1.0%) were also shown in Figure 6. It could be observed that emulsion droplet size elevated from 0.65 μm to 4.05 μm while oil fraction ranging from 0.45 to 0.65, which was also the same as the results of Li’s [36]. This phenomenon mainly was associated with the fact that a particular concentration of CS-PA-CD nanoparticles could only stabilize limited areas on the oil in water interface, therefore, forming larger droplets with smaller total region to attain adequate surface coverage of all droplets. However, the creaming index values were at opposite trend to the droplet size of Pickering emulsions, which was similar to the results of Yang and Su et al. [45]. The microstructure of Pickering emulsions at different oil fractions was illustrated in Figure 7b, showing the same tendency as found in Figure 6b. A similar phenomenon was also reported by Yang and Su et al. [45]. The zeta potential presented no clear tendency corresponding with the trend of droplet sizes and the creaming index values, demonstrated that there was no correlation with the stability of Pickering emulsions. These findings were the same as the effect of particle concentrations.

#### 3.2.2. Rheological Properties of Pickering Emulsions

**Figure 8:**
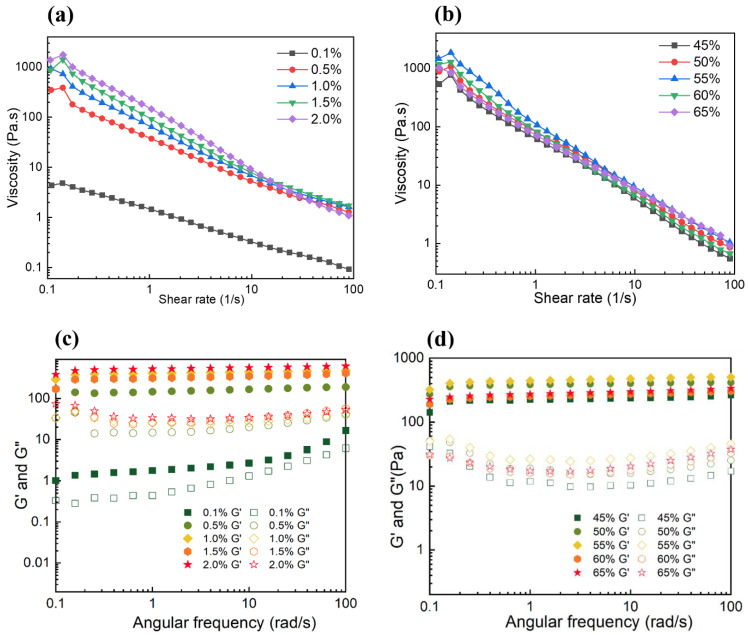
Apparent viscosity (**a**,**b**) and the dynamic rheological behavior (**c**,**d**) of Pickering emulsions under different particle concentrations (**a**,**c**) and oil fractions (**b**,**d**).

The influence of particle concentration on the rheological properties of Pickering emulsions was shown in Figure 8. The rheological properties were characterized by steady-state flow and dynamic oscillatory measurements. The apparent viscosity of all emulsions decreased with the increasing of shear rate from 0.1 to 100 s^−1^, indicating a shear-thinning behavior, which proved that the Pickering emulsions were typical non-Newtonian fluids. It was mainly due to the deformation and disruption of aggregation between droplets [13]. Moreover, the apparent viscosity of Pickering emulsions increased as the particle concentration increased from 0.1% to 2.0%, related to the addition of CS-PA-CD as a thickener to improve the apparent viscosity. The value of G′ was obviously higher than G″ for all samples, revealing the elastic gel-like structure of emulsion gels. Both G′ and G″ slightly increased with increased frequency. These results were similar to the results of Hu [46]. Furthermore, the increasing trend of the value of G′ and G″ demonstrated the enhanced gel strength, corresponding to the reduction of droplet sizes.

The rheological performance at different oil fractions was also presented in Figure 8. The steady-state flow shows similar trend with the influence of particle concentration, where apparent viscosity declined as the shear rate ranging from 0.1 to 100 s^−1^, indicating shear thinning behaviors attributed to the deflocculation of oil droplets. The apparent viscosity was also increasing with the increasing of oil fraction, which Qiu et al. had also found [47]. However, it has a decreased trend when the oil fraction was higher than 55%. Furthermore, this phenomenon appeared in the characterization of the influence of oil fractions on the dynamic oscillatory as well. A similar result was noticed by Hu et al. [46], Ge et al. also found this phenomenon [48]. Hu et al. concluded that higher oil fractions emulsions with relatively lower viscosity could be attributed to phase inversion arising and the formation of unstable W/O emulsions.

### 3.3. Effects of Ionic Strength on Pickering Emulsions Stability

**Figure 9:**
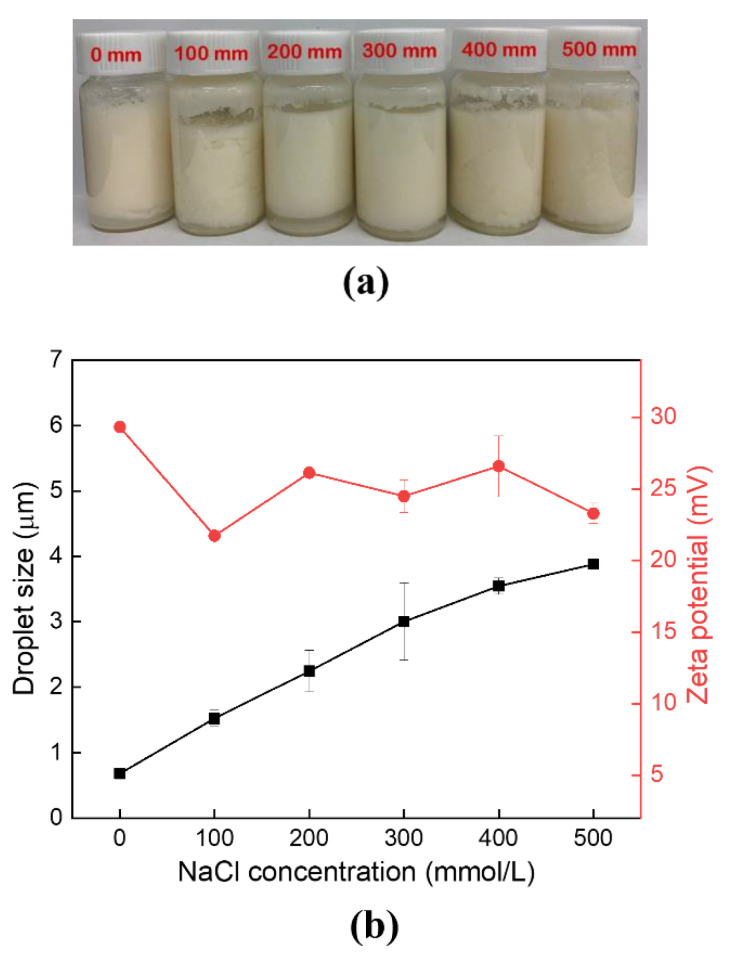
Effects of ionic strength on appearance (**a**) and droplet size (**b**) of Pickering emulsions stabilized by CS-PA-CD nanoparticles.

The ionic strength was one of the environmental influencing factors for food emulsions, which is found to affect the charged particles at the interface between particles. Pickering emulsions stabilized with CS-PA-CD nanoparticles under different NaCl concentrations was estimated in Figure 9. The visual appearance showed little change in Figure 9a, indicating a better ionic stability of Pickering emulsions. However, the emulsions droplet size had significant changes with the change of NaCl concentrations, showing an increasing trend when the NaCl concentration increased, suggesting droplets aggregation. Meanwhile, the zeta potential slightly decreased as the NaCl concentration were increasing and the droplet size were increasing, mainly due to the electrostatic repulsion reduction. All samples were below 30 mV, suggesting non-relation between stability and zeta potentials of Pickering emulsions stabilized with CS-PA-CD nanoparticles, as already confirmed before.

### 3.4. Effects of Temperature on Pickering Emulsions Stability

**Figure 10:**
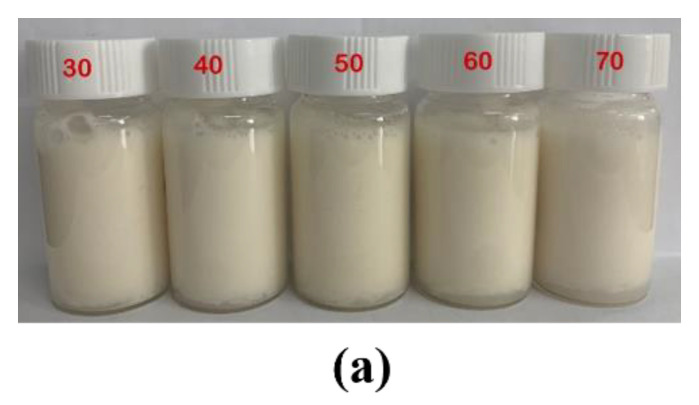

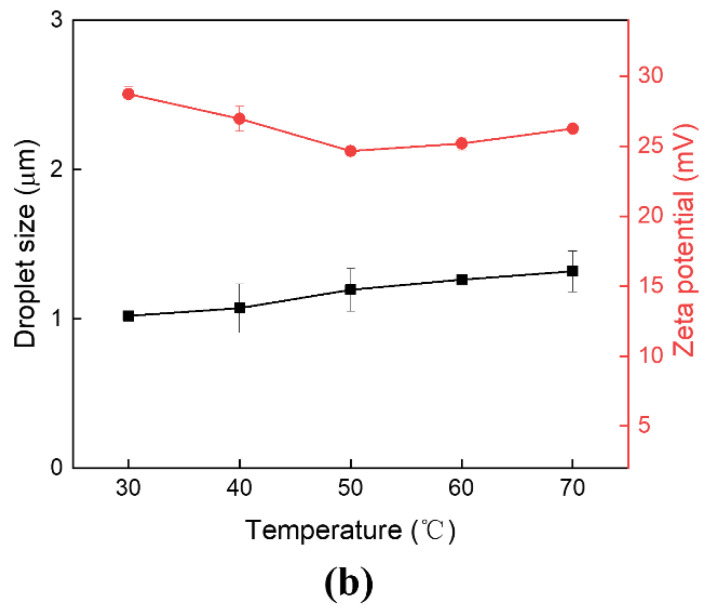
Effects of temperature on appearance (**a**) and droplet size (**b**) of Pickering emulsions stabilized by CS-PA-CD nanoparticles.

The Pickering emulsions were bathed in water at different temperatures for 30 min and then kept at room temperature to investigate its thermal stability. Figure 10 illustrated the Pickering emulsions stabilized by CS-PA-CD nanoparticles under different temperatures. The visual appearance remained unchanged at temperature ranging from 30 to 70 °C, same with the result of droplet sizes, demonstrating the good thermal stability of Pickering emulsions. This finding was similar to the research of Qiu et al. and Dai et al. [47,49]. As shown in Dai’s research, the droplet size of Pickering emulsions stabilized by composite zein-propylene glycol alginate particles appeared no big difference when temperature under 70 °C. Qiu et al. also reported that the droplet sizes of emulsion stabilized by cyclodextrin-based metal-organic frameworks and glycyrrhizic acid showed unchanged up to 70 °C, indicating that emulsions remain stable against thermal treatment.

### 3.5. Effects of pH Value on Pickering Emulsions Stability

**Figure 11:**
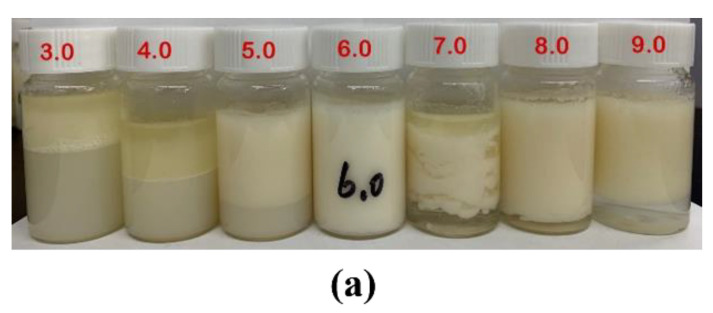

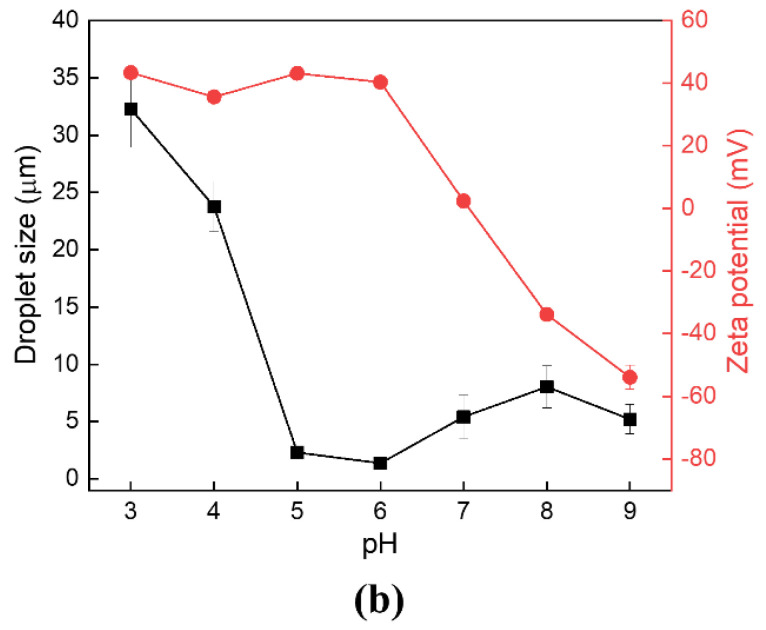
Effects of pH value on appearance (**a**) and droplet size (**b**) of Pickering emulsions stabilized by CS-PA-CD nanoparticles.

The Pickering emulsions under different pH values were shown in Figure 11. At low pH, the Pickering emulsions were unstable regarding to the greater electrostatic repulsions (see Figure 11a) between the polymer chains, thus thinning of the interfacial film due to the dissolution of the particles [9,11]. The Pickering emulsions were highly stable against droplet coalescence and creaming at pH 6, which was attributed to the decrease in electrostatic repulsions (see Figure 5) because of the positive charges of CS neutralize at alkaline pH values, thus nanoparticles can better cover the surface of the oil droplets. The observation was similar to the results of Liu’s [8]. Their findings showed that emulsions stabilized by CS nanoparticles formed at pH < 6.5 were unstable. At pH 6.0–6.5, chitosan nanoparticles have a relative hydrophilic surface due to the partial deprotonation of amines and are a weak particulate emulsifier for O/W Pickering emulsions. However, the emulsions stabilized by chitosan aggregates formed at pH > 6.5 were stable for 2 months and the floccular precipitates formed at pH > 9.0 could also stabilize Pickering emulsions.

## 4. Conclusions

In summary, food-grade O/W Pickering emulsions stabilized by novel CS-PA-CD nanoparticles were prepared. The smallest average size of CS-PA-CD nanoparticle was 434.3 nm and it increased as the chitosan portion increased, and the zeta potential decreased with the increasing of pH value due to its surface amino groups. The successfully cross-linking between phytic acid, chitosan and β-cyclodextrin made it a desirable stabilizer for O/W Pickering emulsions. The droplet size was influenced by particle concentrations and oil fractions. The emulsion emulsified by CS-PA-CD nanoparticles presented a stable state, and the droplet sizes presented no clear tendency corresponding with the trend of zeta potential, attributed to the resistance to coalescence provided by solid particles after the CS-PA-CD nanoparticles are absorbed at the oil–water interface [46]. The rheology measurement confirmed the shear-thinning behavior and the elastic gel-like structure. The ionic strength highly affected emulsions while the temperature affected little. This study presented a new strategy for food-grade Pickering emulsions preparation to expand the application of chitosan and cyclodextrin, which can be supplied in food, cosmetics, and pharmaceutical fields.

## Figures and Tables

**Table 1:** Preparation of CS-PA-CD nanoparticles.

CS Concentration *(m*/*v*)	Acetic Acid (*v*/*v*)	PA-CD Concentration (*m*/*v*)	PA-CD:CS Ratio	Reacting Solution:Ethanol (*v*:*v*)
1.0%	2%	1.0%	1:1	1:10

**Table 2:** Particle size and zeta potential of CS-PA-CD nanoparticles.

PA-CD:CS Mass Ratio	Particle Size (nm)	PDI	Zeta Potential (mV)
2:1	434.2 ± 2.5 ^e^	0.220 ± 0.027 ^ab^	+15.25 ± 3.81 ^c^
1.5:1	481.2 ± 3.0 ^d^	0.211 ± 0.011 ^b^	+11.18 ± 1.38 ^d^
1:1	504.9 ± 11.6 ^c^	0.210 ± 0.010 ^b^	+15.48 ± 1.25 ^c^
1:1.5	612.4 ± 11.1 ^b^	0.247 ± 0.027 ^a^	+21.44 ± 2.00 ^b^
1:2	811.1 ± 7.0 ^a^	0.219 ± 0.005 ^ab^	+26.81 ± 1.84 ^a^

Mean values ± standard deviations. Values with different letters within the same column are significantly different (*p* < 0.05) and the activity order is a > b > c > d > e.

## Data Availability

Not applicable.
